# Oleic Acid and Succinic Acid: A Potent Nutritional Supplement in Improving Hepatic Glycaemic Control in Type 2 Diabetic Sprague–Dawley Rats

**DOI:** 10.1155/2024/5556722

**Published:** 2024-06-19

**Authors:** Kemmoy G. Lattibeaudiere, Ruby Lisa Alexander-Lindo

**Affiliations:** ^1^School of Natural and Applied Sciences, Faculty of Science and Sport, University of Technology, Kingston, Jamaica; ^2^Basic Medical Sciences, Faculty of Medical Sciences, The University of the West Indies, Mona, Kingston, Jamaica

## Abstract

Nutritional supplements are gaining traction for their effects in mitigating the impacts of various health conditions. In particular, many supplements are being proposed to reduce the impacts of type 2 diabetes (T2D), a metabolic condition that has reached global epidemic proportions. Recently, a supplement of oleic acid (OA) and succinic acid (SA; 1 : 1, w/w) was reported to improve glycaemic control in type 2 diabetic (T2D) Sprague–Dawley (S-D) rats through ameliorating insulin release and sensitivity. Here, we investigate the effects of the supplement (OA and SA) on hepatic and pancreatic function in T2D S-D rats. Eighteen (18) S-D rats were rendered diabetic and were divided into three equal groups: diabetic control, diabetic treatment, and diabetic glibenclamide. Another 12 S-D rats were obtained and served as the normal groups. The animals were treated daily with the vehicle, OA and SA (800 mg/kg body weight (bw); 1 : 1), or glibenclamide (10 mg/kg bw) which served as the positive control. The findings indicated that treatment with the supplement resulted in a 35.69 ± 4.22% reduction (*p*=0.006) in blood glucose levels (BGL). Analysis of hepatic enzymes depicted that the nutritional supplement reduced the activity of the gluconeogenesis enzyme, glucose-6-phosphatase (G6P) while improved the activity of catabolic enzymes such as glucose-6-phosphate dehydrogenase (G6PD) and pyruvate kinase (PK). Furthermore, the supplement attenuated oxidative stress through restoration of catalase (CAT) and superoxide dismutase (SOD), while reducing malondialdehyde (MDA) levels. Finally, the supplement showed no liver or kidney toxicity and improved the size and number of pancreatic islets of Langerhans, indicating its potential application in treating T2D. The study highlighted that a supplement of the two organic acids may be beneficial in reducing the rate of pathogenesis of type 2 diabetes. Therefore, it may offer therapeutic value as a dietary or nutritional supplement in the approach against diabetes and its complications.

## 1. Introduction

There has been a trend in the increase of type 2 diabetes (T2D) in both developing and developed societies [1, 2]. With the rate of increase in prevalence and incidence, the metabolic syndrome has reached epidemic proportions with over 422 million persons suffering from the disease [3]. This number is predicted to double over the next decade, accounting for more than 4.4% of world's population. T2D, in particular, accounts for approximately 90% of all cases of diabetes and is generally characterized by initial insulin insensitivity followed by destruction of beta-cells of the pancreas [4–7]. Despite the availability of oral synthetic agents that ameliorate several of these conditions, there is still a high demand for novel therapeutics with higher efficacy and fewer side effects.

In our previous study, it was highlighted that oleic acid (OA) and succinic acid (SA) found in the plant *Desmodium canum* synergistically improve glycaemic control in diabetic S-D rats [8]. In this study, an improvement in insulin sensitivity was documented and played a pivotal role in alleviating the exacerbated state of hyperglycaemia. The individual benefits of the organic acids in the treatment of T2D are well documented with a wealth of supporting evidence [9–12]. In earlier studies, Saravanan and Pari [13] reported on the insulinotropic nature of SA and its esters and proposed the monomethyl ester to be a novel therapeutic agent in the fight against diabetes. Other research confirmed the mechanism of action of the dicarboxylic acid which includes a stimulation of the mevalonate pathway [14–16]. Similarly, the literature purports the beneficial effects of OA in the treatment of T2D. Palomer et al. [17] documented that OA mitigates against insulin resistance and decreases the deposition of diacylglycerides in peripheral tissues.

In the current study, the effects of a supplement of the two organic acids on hepatic glucose metabolism and morphology of pancreatic tissue in diabetic rats are highlighted. The role of the liver in glycaemic modulation is well documented, especially through regulation of gluconeogenesis, glycolysis, and glycogenolysis [18, 19]. Diabetes mellitus disrupts the tight regulation of these processes at both the transcriptional and translational levels. One of the leading factors that disrupts this homeostasis is insulin resistance, which negates the suppressive effects of insulin on gluconeogenesis and glycogenolysis. The expression and activity of gluconeogenetic regulatory enzymes such as glucose-6-phosphatase (G6P) and fructose-1, 6-bisphosphatase (FBP) are significantly elevated in T2D, consequently resulting in an elevated level of glucose hepatic output [20–24]. This partially explains the aberrant glucose levels reported in type 2 diabetics. Other studies have underscored the importance of maintaining a normal gluconeogenic rate in diabetics [25, 26]. Any agent that demonstrates potential to regulate this, whether at the transcriptional level or post-translation levels, may offer therapeutic benefits in glycaemic control.

Moreover, oxidative stress is well documented to be a contributing factor in the progression of diabetes mellitus [27, 28]. An increase in the production of reactive oxygen species (ROS) is linked to the glycation of endogenous antioxidants as well as an increased production of ROS from the mitochondria. Studies have highlighted the role that reactive species play in the degradation of the pancreas and its exacerbation of insulin resistance [27]. These are contributing factors towards the progressive nature of diabetes and ultimately results in the need for therapeutic agents to achieve glycaemic control. Furthermore, the ROS effect on hepatic function is well described in the literature [29–31]. Studies have reported a marked elevation of biomarkers for oxidative stress such as malondialdehyde (MDA) and 8-hydroxy-2′-deoxyguanosine, while decreasing the body's endogenous antioxidants such as catalase (CAT), glutathione, and superoxide dismutase (SOD) [27, 32, 33]. Oxidative stress disrupts the metabolic processes within the liver including those important for glycaemic control. Consequently, increased oxidative stress acts as a progressive factor in exacerbating diabetes and complications associated with the metabolic syndrome. Novel antidiabetic therapeutics should, therefore, be able to mitigate this contributing factor.

Given the antidiabetic nature of a supplement containing both OA and SA, it warrants studying the effects on the hepatic carbohydrate metabolizing enzymes, along with any other effects that the supplement may have on liver function in type 2 diabetic S-D rats. Furthermore, given the importance of the pancreas, examining the potential amelioration of degeneration of the pancreas is noteworthy. It is being hypothesized that a synergy of the two organic acids will improve glycaemic control through an improvement if hepatic and pancreatic functioning.

## 2. Materials and Methodology

### 2.1. Materials

Enzymes used in the study were purchased from Sigma Aldrich, USA. Additionally, D-mannitol, adenosine 5-diphosphate sodium, nicotinamide adenosine dinucleotide (phosphate), glucose-6-phosphate, D-fructose-1,6-bisphosphate trisodium salt, phosphoenolpyruvate, polysorbate 20 (Tween 20), streptozotocin (STZ), succinic acid (bioXTra, >99%, catalogue number: S3674), oleic acid (bioreagent, >99%, catalogue number: O1383), hydrogen peroxide (30%), and pyrogallol were also procured from Sigma Aldrich Co., while inorganic salts (ACS grade) and buffer reagents were purchased from Fisher Scientific, USA. Total bilirubin, serum creatinine, serum creatinine, serum aspartate aminotransferase, and serum alanine aminotransferase kits were purchased from Cayman Chemical Company while LDL-C and HDL-C kits were purchased from Crystal Chem Inc.

### 2.2. Ethical Consideration

The Faculty of Medical Sciences Mona Campus Research Ethics Committee, UWI Mona Ethic Committee granted ethical approval for the use of S-D rats in the study (Ethical approval number: AN07, 2018). The ethical guideline for their usage was strictly followed.

### 2.3. Diabetic Induction

Thirty S-D rats (15 males and 15 females, mean weight of 155 ± 12 g) were retrieved from the Department of Basic Medical Sciences Animal House, UWI, Mona campus. The animals were subsequently randomly divided into five groups, with a sample size of 6 each.

Diabetes induction was done using a method described by [5, 34]. The normal groups (1 and 2) were fed water *ad libitum* for two weeks, after which, they were subjected to an intraperitoneal (ip) injection of citrate buffer (0.3 mL, pH 4.5). This buffer served as the vehicle for STZ in the diabetic groups (3 to 5). In the diabetic induction, these animals were fed a 10% fructose solution for two weeks *ad libitum*. Subsequently, the animals were rendered diabetic through an intraperitoneal (ip) injection of 40 mg/kg bw STZ dissolved in 0.3 mL of cold citrate buffer. Their glycaemic state was assessed by measuring their nonfasted BGL 1 week postadministration. Blood glucose values ≥ 15.6 mM were considered diabetic [35].

The animals were subsequently treated daily with the respective treatments as described below:

  Group 1: Normal Control (NC)-0.3 mL 10% Tween 20
  Group 2: Normal Treatment (NT)-Oleic acid + Succinic acid 800 mg/kg bw (1 : 1, OA + SA) [8].
  Group 3-Diabetic Control (DC)-0.3 mL 10% Tween 20
  Group 4-Diabetic Treatment (DT)-supplement of OA + SA (OA + SA)-800 mg/kg bw (1 : 1, OA + SA)
  Group 5-Diabetic Glibenclamide (DGLIB)-0 mg/kg bw Glibenclamide [8].

Nonfasting blood glucose levels were monitored 1 week post STZ or citrate buffer administration and at the end of the study. The percentage change in BGL was calculated as reported by [36, 37] shown below:

(1) $$\begin{array}{c} \frac{\text{Final BGL}-\text{Initial BGL}\times 100\%}{\text{Initial BGL}} \end{array}$$

### 2.4. Animal Sacrifice

At the end of 28 d of treatment, the animals were anaesthetized through 65 mg/kg bw (ip) of sodium pentobarbital [8, 38]. Blood was collected from the renal arteries and analysed for liver and kidney biomarkers such as bilirubin, alanine aminotransferase, aspartate aminotransferase, creatinine, urea, and blood urea nitrogen. The liver, pancreas, and kidneys were harvested, rinsed in distilled water, and then dried before being preserved either by storing at −80°C (liver) or in 10% neutral buffered formalin (pancreas).

### 2.5. Preparation of Liver Homogenate

Liver homogenates were prepared as described by [39] with modifications. A 10% (w/v) homogenate was prepared by homogenizing liver samples in a 10 mM Tris-HCl (pH 7.4) containing 60 mM sucrose, 220 mM mannitol, and 1 mM EDTA. The homogenates were then centrifuged at 4°C at 6000 rpm for 15 min. The supernatants were removed and stored at −80°C for the estimation of protein content by Bradford assay, and the activity of glucose 6-phosphatase (G6P), glucose 6-phosphate dehydrogenase (G6PD), fructose-1,6-bisphosphatase (FBP), pyruvate kinase (PK), malondialdehyde (MDA), catalase (CAT), and superoxide dismutase (SOD). All analyses were done with a sample size of 6.

### 2.6. Analysis of Hepatic Carbohydrate Metabolic Enzymes

The activity of G6P and FBP was determined as described by [40] with minor modifications. A mixture of citrate buffer (0.1 M, pH 6.6, 3 mL) and 1 mL of glucose 6 phosphate disodium salt (200 mM) were preheated at 37°C for 5 min. The liver homogenate was then added (0.1 mL) and further incubated at 37°C for 5 min. The resulting inorganic phosphate was developed using Taussky-Shor colour reagent and read at 600 nm using a Thermo Scientific GENESYS 30 (model no: 9A1X358128) UV/Vis spectrophotometer. In the analysis of FBP, a reaction mixture was prepared which contained 2.74 mL of glycine buffer (100 mM, pH 9.5), a solution of fructose 1,6-bisiphosphate solution (0.10 mL, 41 mM), manganese chloride solution (0.02 mL, 51 mM), phosphoglucose isomerase enzyme solution (catalogue number: P9455; 0.02 mL), G6PD enzyme solution (catalogue number: G7787-1KU, 0.01 mL), NADP^+^ (0.10 mL, 12 mM), and liver homogenate solution (0.2 mL). The mixture was incubated for 5 min and then absorbance readings were read at 340 nm.

The analysis of PK was done as described by Storey and Bailey [41] with modifications. A reaction mixture of 3.0 mL containing 5 mM phosphoenol pyruvate, 5 mM ADP, 0.2 mM NADH, 10 mM MgCl_2_, 100 mM KCl, and 14 U of lactate dehydrogenase (catalogue number: 427217-M). The reaction was initiated following the addition of 50 *μ*L of a 5-fold diluted liver homogenate. Absorbance readings were measured at 340 nm using a UV/Vis spectrophotometer at 10 s intervals for 5 min.

G6PD was measured as described by Noltmann, Gubler, and Kuby [42] with modifications. A stock solution containing a final concentration of 20 mM Tris-HCl (pH 7.5), 2 mM glucose-6-phosphate, 0.67 mM NADP^+^, and 10 mM MgCl_2_ was prepared. To this reaction mixture, a 5-fold diluted liver homogenate was added (0.1 mL), and OD readings at 340 nm were monitored at 10 s intervals for 5 min to measure the formation of NADPH.

### 2.7. Hepatic Oxidative Stress

#### 2.7.1. Lipid Peroxidation (Malondialdehdye (MDA))

The method was adapted from [43] with minor modifications. MDA was extracted from liver homogenate using a solution of ferric chloride (100 mM) and ascorbic acid (100 mM). The MDA was then reacted with 0.67% thiobarbituric acid (500 *μ*L) and subsequently centrifuged. The supernatant was analysed using a spectrophotometer at 535 nm against a suitable reagent blank.

#### 2.7.2. Catalase (CAT)

The CAT activity was determined based on the protocol described by [43]. A reaction mixture contained 1.25 mL of 50 mM phosphate buffer (pH 5), 200 *μ*L of 5.8 mM H_2_O_2_, and 70 *μ*L of the homogenate was prepared. The change in the absorbance at 240 nm after 1 min was noted. An absorbance change of 0.001 units/min represents 1 U of catalase activity.

#### 2.7.3. Superoxide Dismutase (SOD)

The activity of superoxide dismutase (SOD) was determined using pyrogallol autooxidation protocol. The reaction mixture (2 mL) consisted of 1 mL of 100 mM sodium phosphate buffer (pH 8.2), 3.3 mM EDTA (62 *μ*L), 60 *μ*L of tissue homogenate diluted 20-fold and distilled water. The reaction was initiated by the addition of 60 *μ*L of freshly prepared pyrogallol (8.1 mM). The change in the absorbance at 420 nm was measured at 30 s intervals for 3 min. The percentage inhibition of autoxidation was calculated, and 1 U of SOD activity is amount of enzyme that is needed to inhibit the autoxidation of pyrogallol by a half [44].

### 2.8. Analysis of Serum Samples

Assay kits purchased from Cayman Chemical Company or Crystal Chem Inc. were used to determine the levels of total bilirubin, creatine, urea, aspartate aminotransferase (AST), alanine aminotransferase (ALT), low-density lipoprotein cholesterol (LDL-C), and high-density lipoprotein (HDL-C) within rats' sera. Blood samples were retrieved from the renal arteries, stored in vacutainers, and allowed to clot at 25°C for 30 min. The samples were then centrifuged (Labconco Refrigerated Centrifuge) at 2000 × *g* at 4°C and the serum was collected. All serum analyses were done in triplicate.

#### 2.8.1. Total Bilirubin Levels

Total bilirubin levels were assayed using a total and direct Bilirubin kit purchased from Cayman Chemical Company (catalogue no. 701720) with a lower limit of detection (LLOD) and lower limit of quantification (LLOQ) of 0.045 mg/dL and 0.25 mg/dL, respectively. Briefly, bilirubin assay catalyst (100 *μ*L) was added to wells of a 96 well microplate, to which samples/standard were added (50 *μ*L) and left to incubate at room temperature for 10 min in the dark. Signal and background reagents were prepared and added to respective wells followed by gentle pipetting. The samples were incubated for a further 15 min, followed by the addition of a total bilirubin probe (75 *μ*L) and then read using a microplate reader (Agilent BioTek) at 600 nm.

#### 2.8.2. Serum Creatinine Levels

Serum creatinine levels were determined using a Rat Creatinine Assay Kit purchased from Crystal Chem Inc. (catalogue number: 80340, sensitivity: 0.15 mg/dL) based on the manufacturer's instructions. Samples/standards (8 *μ*L) were added to microplate wells to which 270 *μ*L of sarcosine oxidase solution were added. The mixture was incubated at 37°C for 5 min and read at 550 nm. This was followed by the addition of 90 *μ*L of peroxidase solution and further incubation at 37°C for 5 min, and subsequently rereading at 550 nm.

#### 2.8.3. Serum Urea

Rats' serum urea was determined using the assay kit purchased from Cayman Chemical Company (catalogue number: 700620) which has a precision between 1.7% and 2.1% and a LLOD of 0.05 mM. A series of urea standards was prepared and 20 *μ*L of each was added to individual wells in a microplate, to which 150 *μ*L of diluted buffer were added. The procedure was repeated with serum samples. The reaction was initiated by the addition of 20 *μ*L of urease and then the mixtures were incubated for 10 min at room temperature. Subsequently, ammonia detector reagent (10 *μ*L) was added to each well and the microplate incubated for 15 min at room temperature. Finally, the fluorescence was read at excitation wavelength of 405 nm and an emission wavelength of 480 nm. Blood urea nitrogen (BUN) was calculated by dividing the serum urea by a factor of 2.14.

#### 2.8.4. Serum Aspartate Aminotransferase (AST) and Alanine Aminotransferase Kits (ALT)

Sample AST levels were measured using an assay kit purchased from Cayman Chemicals Company (catalogue number: 701640; LLOQ: 0.01 U/mL and precision: 4.1%). Briefly, sample wells were prepared by the addition of 150 *μ*L of AST substrate, 20 *μ*L of AST cofactor, and 20 *μ*L of samples. Standard was prepared in a similar fashion where the sample was substituted with 20 *μ*L of standard. The mixtures were incubated at 37°C for 15 min, followed by the addition of the AST initiator. Samples and standards were immediately read at 340 nm once per min for 10 min.

ALT levels were determined in a similar manner using ALT substrate, ALT cofactor, and ALT initiator as described by the assay kit purchased from Cayman Chemical Company (catalogue number: 700260; LLOD: 0.006 U/mL and precision: 5.8%).

#### 2.8.5. Serum HDL-C, LDL-C, Total Cholesterol (TC), and Triacylglycerol (TAG)

HDL-C (catalogue number: 79970; sensitivity: 1.1 mg/dL) and LDL-C (catalogue number: 79960; sensitivity: 4.5 mg/dL) assay kits were purchased from Crystal Chem Inc. and used to determine the levels of HDL-C and LDL-C, respectively. In the assays, serum samples (3 *μ*L) were mixed with 225 *μ*L of polyvinyl sulfonic acid (PVS)/polyethylene-glycol methyl ether (PGME) and left to incubate at 37°C for 5 min. Subsequently, 75 *μ*L of enzymatic solution was added and the mixtures incubated at 37°C for 5 min then read at 600 nm against a series of HDL-C standard. In the LDL-C assay, serum samples (3 *μ*L) were mixed with PVS/PGME and incubated at 37°C for 5 min. After which, 75 *μ*L of decomplexing reagent was added and the mixtures incubated at 37°C for 5 min then read at 600 nm against a series of LDL-C standard.

#### 2.8.6. Total Cholesterol

The total cholesterol in rats' sera was estimated using Zak's method [45]. Serum (0.1 mL) was added to 4 mL of FeCl_3_ precipitating reagent. The mixture was shaken on an automated shaker for approximately 3 min, after which, it was filtered using Whatman number 41 filter paper. To 3 mL of the filtrate, 2 mL of concentrated sulfuric acid was added. The solution was mixed and allowed to stand at room temperature for 10 min. The resultant red-violent solution was read at 560 nm using a UV/Vis spectrophotometer against a suitable reagent blank. A series of cholesterol standards was also prepared and read at 560 nm. The data was used to obtain a standard curve which was used to estimate the TC in the unknowns. TAG was calculated by rearranging Friedewald's formula: LDL-C = (TC) − (HDL-C) − (TG/5) [46].

### 2.9. Histopathological Analysis of Pancreas

Pancreatic tissues were harvested, washed in cold saline, and then weighed. These were then fixed in 10% neutral buffered formalin and were subsequently dehydrated using a series of ethanol treatment. The tissues were subsequently embedded in paraffin wax and sectioned into 10 *μ*m thick segments using a microtone. The tissues were stained using haematoxylin and eosin and analysed using a Nikon Eclipse E 200 light microscope at ×40 objective lens. The number of islets of Langerhans in ten random microscope fields were counted and documented while the diameters of the islets were estimated using ImageJ software bundled with 64 bit Java 1.8.0_172.

### 2.10. Statistical Analysis

Data were expressed as mean ± standard error of the mean and analysed using IBM SPSS Statistics for Windows, version 27 (IBM Corp., Armonk, NY, USA). Comparisons among the groups were carried out using one-way analysis of variance (ANOVA), followed by the Tukey post hoc test, and *p* ≤ 0.05 was considered to be statistically significant. Additionally, the magnitude of the significant differences obtained between the D-OA + SA and the DC group was further calculated using Cohen's *d*, with *d* ≥ 0.2 ≤ 0.499 considered a “small effect,” *d* ≥ 0.5 ≤ 0.799 considered a “medium effect,” and *d* ≥ 0.8 considered a “large effect.”

## 3. Results

### 3.1. The Effect of the Nutritional Supplement on BGL in Diabetic and Nondiabetic S-D Rats

The nutritional supplement of OA and SA synergistically improves glycaemic control in diabetic rats as noted in Table 1. Treatment with the supplement resulted in a decrease in BGL from 22.92 to 14.48 mM, accounting for a 35.69 ± 4.22% reduction (*p*=0.006) in blood glucose concentrations. Similar results were observed with the positive control, where glibenclamide resulted in a 39.88 ± 7.55% reduction in BGL of the diabetic animals (*p*=0.004). There was a noticeable 20.10 ± 13.57% increase in the diabetic untreated animal. Though there was no significant difference between the initial and the final data readings for these animals, there is a noticeable trend for increase in glycaemic state, indicating the progressive nature of T2D. Treatment with the supplement, however, had no effect on the normal rats, which may indicate that there is a threshold of BGL for the supplement to provoke a hypoglycaemic response.

### 3.2. Hepatic Enzymes in Carbohydrate Metabolism and Serum Lipid Profile in Diabetic and Nondiabetic S-D Rats

**Figure 1:**
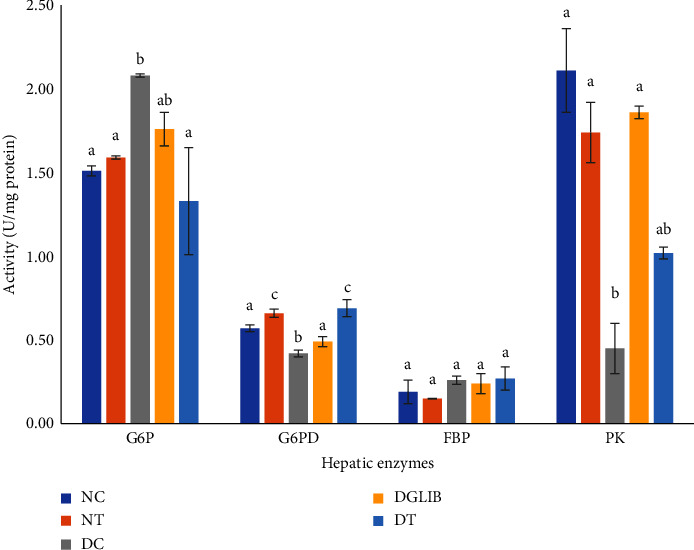
The effect of treatment on the activity of hepatic carbohydrate metabolizing enzymes. Data are expressed as mean ± standard error of the mean and were analysed by Tukey post hoc test (*n* = 6). G6P: glucose 6 phosphatase, G6PD: glucose-6-phosphate dehydrogenase, FBP: fructose-1,6-bisphosphatase, PK: pyruvate kinase, NC: normal control, NT: normal treatment, DC: diabetic control, DGLIB: diabetic glibenclamide, DT: diabetic treatment group, where the letters above the columns represent the following: a: statistically significant differences when compared with the values of the same enzyme superscripts b and c. b: statistically significant differences when compared with the values of the same enzyme superscripts a and b. c: statistically significant differences when compared with the values of the same enzyme superscripts b and c. ab: No statistically significant difference when compared with the values indicated by superscripts a or b for the same enzyme.

As reflected in Figure 1, untreated diabetic animals reported a marked increase in the activity of G6P, reflecting an increased rate of gluconeogenesis. There was also a marked decrease in the activity of PK (0.45 ± 0.15 U/mg protein; *p* < 0.001) and G6PD (0.42 ± 0.02 U/mg protein; *p*=0.043) when compared with the normal control S-D rats (2.11 ± 0.25 U/mg protein and 0.57 ± 0.02 U/mg protein, respectively). On the other hand, the mixture of the two carboxylic acids significantly retarded the activity of the gluconeogenetic enzyme, G6P to levels comparable to those seen in the normoglycaemic rats (1.31 vs 1.51 U/mg protein, *p*=0.341). This was also significantly lower than the DC group with a large size effect as reported by Cohen's *d* of 1.89. Furthermore, the efficacy of the supplement resulted in a large significant increase in the activity of enzymes involved in the catabolic reaction of glucose (G6PD: *p* < 0.05, *d* = 1.73 and PK: *p* < 0.05, *d* = 1.02). However, there was no statistically significant effect reported in the normal animals that were treated with the cocktail of organic acids. The 28 d treatment with glibenclamide demonstrated similar results to that of the animals treated with the naturally occurring organic acids.

**Figure 2:**
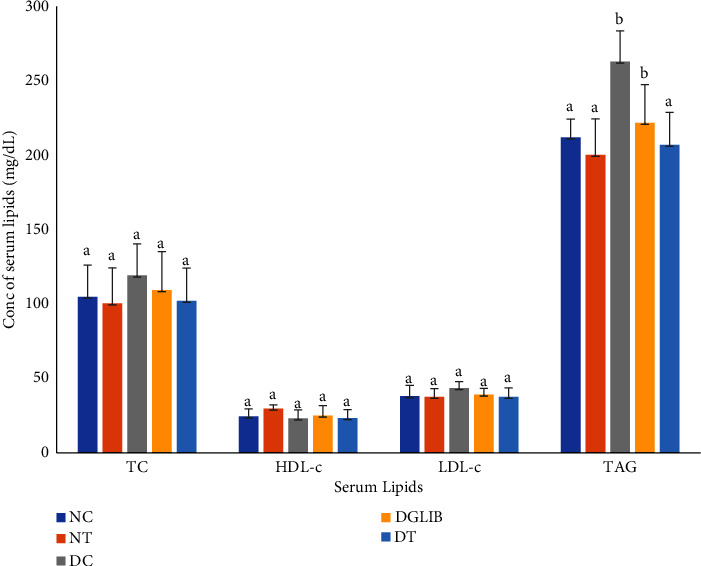
Serum lipids in diabetic and nondiabetic rats fed the nutritional supplements. Data are expressed as mean ± standard error of the mean and were analysed by Tukey post hoc test (*n* = 6). TC: total cholesterol, HDL-C: high-density lipoprotein cholesterol, LDL-C: low-density lipoprotein cholesterol, TAG: triacylglycerol, NC: normal control, NT: normal treatment, DC: diabetic control, DGLIB: diabetic glibenclamide, DT: diabetic treatment group, where the letters above the columns represent the following: a: statistically significant differences when compared with the values of the same parameter with superscript b. b: statistically significant differences when compared with the values of the same parameter with superscript a.

Over the period of the study, there were no differences among the groups for total cholesterol (TC), high-density lipoprotein cholesterol (HDL-C), and low-density lipoprotein cholesterol (LDL-C, Figure 2). However, there was a significant increase in the serum triacylglycerol (TAG) for the diabetic control group when compared with the NC group (262.84 ± 9.41 mg/dL vs 211.97 ± 5.67 mg/dL, *p* < 0.05). Notable, the supplement of OA and SA reduced the serum TAG (206.97 ± 8.35 mg/dL, *p* < 0.05 and *d* = 0.68) when compared with the DC group and no significant difference when compared with the NC group.

### 3.3. Toxicological Effects on Renal and Hepatic Function

The analysis of serum biomarkers for liver and kidney function serves to highlight pathological changes in these key regulatory organs due to diabetes and due to the administration of the nutritional cocktail. Statistical analysis established that there were no significant alterations in the biomarkers assayed.

### 3.4. Oxidative Stress in Hepatic Tissues

**Figure 3:**
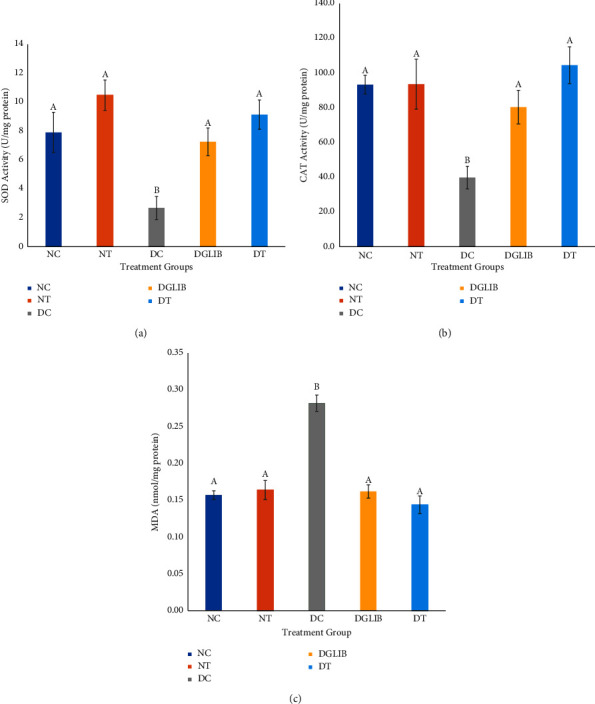
(a) The effect of the various treatments on the activity of superoxide dismutase. (b) The effect of the treatment options on catalase activity in diabetic and normal S-D rats. (c) The effect of the various treatments on the concentration of hepatic MDA in normal and diabetic Sprague–Dawley rats. All data are expressed as mean ± standard error of the mean and were analysed by Tukey post hoc test (*n* = 6). NC: normal control, NT: normal treatment, DC: diabetic control, DGLIB: diabetic glibenclamide, DT: diabetic treatment group, where the letters above the columns represent the following: A: statistically significant differences when compared with the bars with letter B. B: statistically significant differences when compared with the bars with letter A.

Oxidative stress augments many of the complications associated with diabetes mellitus. An increased level of hepatic oxidative stress is linked to a myriad of complications observed in diabetics. The study highlighted this through an increased levels of reactive oxygen species within the hepatic tissues with a decrease in endogenous antioxidant enzymes such as SOD shown in Figure 3(a) (7.91 ± 1.40 vs 2.68 ± 0.80 U/mg protein; *p* < 0.001) and CAT in Figure 3(b) (93.33 ± 6.21 vs. 39.82 ± 8.41 U/mg protein; *p* < 0.001) when compared with the NC group. Similarly, the presence of high levels of MDA in the hepatic tissues of the DC group further supported the increased level of ROS present within the tissues. Treatment with the supplement reversed this with a large significant improvement of CAT (104.54 ± 12.84 U/mg protein, *p*=0.028, *d* = 1.39) and SOD (9.14 ± 1.01, *p*=0.001, *d* = 1.01) activities, ultimately preventing the oxidative degradation of lipids, and the formation of MDA (0.14 ± 0.012, *p* < 0.001, *d* = 1.08). A similar result was observed with the glibenclamide-treated group, exhibiting no significant differences when compared with the NC group (Figures 3(a)–3(c)).

### 3.5. Alterations in the Structure, Number, and Size of the Islets of Langerhans

Table 2 depicts a significant reduction in the number of islets of Langerhans in all diabetic groups. This was achieved through the destruction of the beta cells of the pancreas by the STZ administered. The DC group showed the lowest number of islets with 1.00 ± 0.26 vs 3.88 ± 0.48 for the NC group. Though the supplement failed to increase the number of islets, it promoted a significant restoration of the size of the islets (312.53 ± 23.60 vs. 383.61 ± 35.53 *μ*m, respectively, *p* = 0.142) when compared with the NC group. The supplement also showed a large significant increase in the size of the islets of Langerhans when compared with the DC group (*p* = 0.048, *d* = 1.07). On the other hand, glibenclamide failed to restore the size of the islets for its respective diabetic group.

Histopathological analysis of the pancreatic tissues highlighted normal architecture of the pancreatic acinar cells and islets of Langerhans in the NC (A) and NT (B) groups. However, significant atrophy of the islets of Langerhans of the DC (C) and DGLIB (D) groups was observed (Table 2). Additionally, necrosis was evident in all the diabetic groups (C, D, and E) with more extensive necrosis being observed in the DC group with more eosinophilic cells, alluding to destruction of the nuclei of these cells. Treatment with the nutritional supplement resulted in less necrotic damage being observed in the DT group (E).

The normal architecture of the acinar cells (denoted by the letter A) and the islets (denoted by the letter I) is shown in the NC and NT groups. However, the diabetic untreated group (DC) showed changes in the morphology of the islets with a reduction of stained nuclei along with necrosis (represented by arrows), and with atrophy of the endocrine cells. The DGLIB animals also had distorted and atrophic endocrine cells which is consistent with diabetes, while in the DT group, there was only mild necrosis observed in the endocrine portion of the pancreatic tissues.

## 4. Discussion

End stage diabetes affects the liver adversely and reduces its role in maintaining homeostasis in carbohydrate metabolism. This paper sought to investigate the effect that a nutritional supplement of OA and SA has on liver function in type 2 diabetic rats. As previously documented, the supplement possessed antidiabetic effect and showed a 35.69 ± 4.22% reduction in BGL. The pharmacological effect being reported stemmed from the improvement in insulin sensitivity as described in our previous paper [8]. In addition to improve absorption of glucose into peripheral tissues, improved responses to insulin have been reported to suppress the secretion of glucagon from the *α*-cells of the pancreas and ultimately reduced hepatic gluconeogenesis.

An examination of some of the hepatic enzymes involved in carbohydrate metabolism suggested an overall elevation in the activity of glucose-6-phosphatase (G6P) in the diabetic control group. G6P plays a pivotal role in the synthesis of glucose in the gluconeogenesis and any increase in this enzyme, typically translates to an increase in the rate of gluconeogenesis [21, 22]. Several studies have documented that an increased hepatic glucose output contributes to the increased hyperglycaemic state and the current findings supported this. An elevation of the rate of gluconeogenesis was observed through an increase in the activity of G6P (2.08 ± 0.01 vs. 1.51 ± 0.03 U/mg protein, *p* < 0.05 when compared with the NC group). The increase in gluconeogenesis partially explains the 20.10 ± 13.57% increase in BGL as documented in Table 1. In comparison to this, the nutritional supplement significantly normalized the levels of the gluconeogenetic enzyme. This may have been achieved through the inhibition of expression of the enzyme at the transcription level. Free fatty acids have been documented to inhibit the expression of peroxisome-proliferator-activated receptor-*γ* coactivator 1*α* (PPAR*γ*-1 *α*), a transcription cofactor necessary for the expression of gluconeogenetic enzymes [47]. The nutritional supplement consists of OA, resulting in an increase in the circulating free fatty acids, thus reducing the expression of PPAR*γ*-1 *α*. Moreover, the supplement increased the expression of G6PD, a regulatory enzyme in the pentose phosphate pathway. The literature reports that hyperglycaemia reduces the activity of the enzyme through phosphorylation and thus inactivation of the enzyme [48, 49]. The exacerbated state of glycaemia reported in the DC animals, therefore, accounted for the low activity of G6PD (0.42 ± 0.02 U/mg protein) observed. Successful reduction in the rate of gluconeogenesis is a hallmark for ameliorating the hyperglycaemic state of T2D, indicating the antidiabetic nature of the supplement. However, there were no differences in the enzymes assayed in the NT group when compared with the NC animals, further suggesting the specific nature of the supplement. That is, the supplement induces an antihyperglycaemic nature when BGLs are high.

The nutritional supplement also improved oxidative stress within the hepatic tissues. The correlation between diabetes and oxidative stress is well documented, where the elevation of reactive oxygen species (ROS) is responsible for several of the complications associated with the metabolic disease. These include insulin resistance, a condition that is well documented to be consistent with T2D, as well as liver diseases and associated coronary heart diseases [50, 51]. As previously reported, the supplement resulted in an increase in the activity of G6PDH, hence an increase in the production of NADPH. The coenzyme played a central role in the promotion and regeneration of reduced glutathione, a notorious antioxidant molecule. The reducing power from NADPH may indirectly influence the restoration of the activity of CAT and SOD documented within the diabetic groups treated with the supplement and the positive control, glibenclamide. Moreover, the levels of malondialdehyde, a marker for lipid peroxidation were normalized in both the aforementioned groups when compared with the diabetic untreated group. With significant changes in the measured parameter within the diabetic untreated group, there is clear evidence of oxidative stress. The nutritional supplement protected the hepatic tissues from destruction by ROS, possibly through a reduction in the formation of advanced glycated end products (AGEs) that can bind and inactivate antioxidants such as SOD and CAT. A decrease in AGEs would explain the normalized activities of the antioxidant enzymes measured. Other possible mechanisms include a reduction in the production of ROS due to the hypoglycaemic property of the supplement. Studies have confirmed a positive correlation between the BGL and the levels of ROS; thus, any reduction in BGL may be followed by a reduction in ROS levels [27, 52].

Overall, a reduction in oxidative stress within the liver is advantageous in maintaining the integrity of the hepatocytes and the role they play in maintaining glycaemic homeostasis and detoxification. Moreover, there was no reported hepatic or renal toxicity due to the supplement. The evidence of this lies within the biomarkers that were monitored, where there were no differences among the groups. Generally, xenobiotics enter the liver through the hepatic portal vein and eventually to hepatocytes, where they may exert toxic effects [53, 54]. This often leads to an elevation of serum total bilirubin, alanine amino transferase, aspartate amino transferase, among others. The study underscored that the supplement had no effect on these hepatic biomarkers (Table 3), indicating that there was no extensive damage done to the liver tissues. Similarly, many xenobiotics cause damage to renal cells and therefore affect the glomerular filtration rate. Consequently, there may be an elevation in metabolites that are filtered by the kidneys. In this study, we examined the serum levels of creatinine, urea, and blood urea nitrogen, none of which displayed an elevation due to the cocktail of organic acids. These tests confirm that the supplement had low toxicity and may therefore form a novel therapeutic approach to T2D.

Further to the effect of the nutritional supplement on liver function, the impact on serum lipid profile and the integrity of pancreatic tissues were highlighted. There was a significant increase in serum triglycerides in the diabetic untreated group, which was normalized in the supplement-treated group. This was possible through regulating the improved liver functions including the metabolism of lipids. A mitigation of glycaemia aids in restoring homeostasis, thus accounting for the reduction of triglyceride. Additionally, OA has been documented to increase metabolism of fats and, therefore, reduce the levels of TAGs [55].

**Figure 4:**
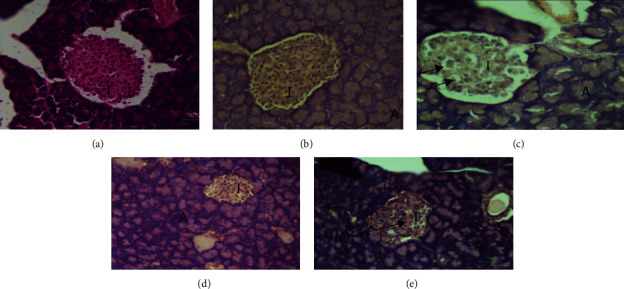
Light photomicrograph of a section of the pancreas exposing an islet of Langerhans (magnification ×400) from each treatment group: (a) NC: normal control, 105 *μ*m, (b) NT: normal treatment, 105 *μ*m, (c) DC: diabetic control, 105 *μ*m, (d) DT: diabetic treatment, 100 *μ*m, (e) DGLIB: diabetic glibenclamide, 102 *μ*m. The letter “A” represents the acinar cells while “I” denotes islets of Langerhans, while 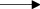 represents possible necrosis.

The nutritional supplement also showed efficacy in protecting the endocrine portion of the pancreatic tissues from degeneration (Figures 4(a)–4(e)). The DC animals had a marked increase in the destruction of the islets, especially cells towards the middle of the islets, where the beta-cells of the pancreas are found (Figure 4(c)). There was a noticeable increase in cytoplasm vacuolation, eosinophilia, and pyknosis of the nuclei, all of which indicate necrosis of the pancreatic tissue (Figure 4(c)). This is possibly as a result of the pharmacodynamics of STZ or oxidative stress. With the level of damages done within the DC groups when compared with the other diabetic groups, it is very likely that much of the damages done were due to oxidative stress. The pancreatic *β*-cells are highly susceptible to ROS due to the limited antioxidants present [56]. Consequently, an elevation in ROS, as was seen in the DC group, may have dire consequences on the pancreatic cells. The supplement, however, prevented this through a reduction in the damage done to the *β*-cells (Figure 4(d)). This resulted in a general increase in size of the islets of Langerhans when compared with all the other diabetic groups, with no significant difference when compared with the normal groups (Table 2). The pancreatic protective role coupled with the reduction of hepatic production of glucose nature due to the supplement was able to increase the secretion of insulin, thus assisting with the reduction of the blood glucose concentrations.

The study, therefore, added further evidence of the previously reported antidiabetic nature of the nutritional supplement. OA and SA synergistically reduced the rate of depletion of the function of the liver and pancreas, both of which are essential for maintaining efficient glucose metabolism. In the DC animals, the dysfunction of these organs contributed to the aberrant glucose levels in the blood, which provoked diabetic symptoms. However, the nutritional supplement ameliorated the condition through a protection or reducing the rate of damage done to these vital organs. Consequently, resulting in an improved metabolism of glucose, and thus resulted in an alleviation of diabetic conditions of the animals.

## 5. Conclusion

The nutritional supplement of OA and SA served as an antidiabetic agent which aided in maintaining the glycaemic regulatory role of the liver. The study underscored that the supplement mediated glycaemic control through a restoration of carbohydrate metabolic enzymes and protected against ROS destruction. The role of the liver in homeostatic control has been well documented and the study established that the supplement offered a protective role against damage in diabetic rats. Furthermore, the supplement also preserved the architecture of the pancreatic tissues and reduced STZ-related necrosis in the islets. This allowed for a successful maintenance in the role of the pancreas and therefore, resulted in an antihyperglycaemic effect of the supplement.

## Figures and Tables

**Table 1:** Change in BGL of diabetic and nondiabetic rats over the period of the study.

Treatment groups	Initial BGL (mM)	Final BGL (mM)	% change in BGL
NC	7.97	5.13	−8.13 ± 7.09
NT	5.73	6.10	−3.96 ± 0.15
DC	19.53	25.69	20.10 ± 13.57
DT	22.92	14.48^*∗*^	−35.69 ± 4.22
DGLIB	23.69	14.35^*∗*^	−39.88 ± 7.55

Data are expressed as mean ± standard error of the mean. BGL: blood glucose levels, NC: normal control, NT: normal treatment, DC: diabetic control, DT: diabetic treatment, DGLIB: diabetic glibenclamide group. ^*∗*^*p* ≤ 0.05 when compared with the DC group.

**Table 2:** The number of islets/10 microscope field and diameter of pancreatic islets of diabetic and nondiabetic animals after 28 d of treatment with their respective regime.

Treatment groups	Number of islets/10 microscope fields	Diameter of islets (*μ*m)
NC	3.88 ± 0.48^a^	383.61 ± 35.53^a^
NT	3.75 ± 0.85^a^	343.12 ± 36.56^a^
DC	1.00 ± 0.26^b^	185.71 ± 6.53^b^
DGLIB	1.40 ± 0.25^b^	245.64 ± 29.80^b^
DT	1.80 ± 0.45^b^	312.53 ± 23.60^a^

All data are expressed as mean ± standard error of the mean and were analysed by Tukey post hoc test (*n* = 6). NC: normal control, NT: normal treatment, DC: diabetic control, DGLIB: diabetic glibenclamide, DT: diabetic treatment group. The superscript ‘a' indicates a significant difference when compared with the superscript ‘b' within the same column, while the superscript ‘b' indicates a significant difference when compared with the superscript ‘a' in the same column.

**Table 3:** The effect of a synergy of oleic acid and succinic acid on hepatic and renal biomarkers.

Groups	Bilirubin (mg/dL)	Creatinine (mg/dL)	Serum urea (mM)	Blood urea nitrogen (mM)	AST (U/L)	ALT (U/L)
NC	1.07 ± 0.04^a^	0.84 ± 0.34^a^	26.47 ± 7.26^a^	12.37 ± 1.70^a^	220.57 ± 26.66^a^	17.12 ± 1.45^a^
NT	0.96 ± 0.02^a^	0.95 ± 0.25^a^	24.54 ± 1.72^a^	11.70 ± 0.81^a^	187.36 ± 41.08^a^	17.83 ± 2.90^a^
DC	1.24 ± 0.19^a^	1.21 ± 0.21^a^	34.87 ± 9.63^a^	16.30 ± 1.97^a^	267.71 ± 21.73^a^	21.09 ± 2.14^a^
DGLIB	1.14 ± 0.07^a^	1.04 ± 0.26^a^	27.87 ± 5.86^a^	13.00 ± 4.29^a^	216.64 ± 41.06^a^	15.04 ± 2.34^a^
DT	1.16 ± 0.04^a^	0.80 ± 0.07^a^	29.58 ± 1.41^a^	13.83 ± 0.66^a^	168.07 ± 25.67^a^	15.35 ± 0.52^a^

Values were expressed as mean ± standard error of the mean (*n* = 6). AST: aspartate aminotransferase, ALT: alanine aminotransferase, NC: normal control, NT: normal treatment, DC: diabetic control, DGLIB: diabetic glibenclamide, DT: diabetic treatment group, where the superscript, “^a^” indicates there were no significant differences among the groups studied.

## Data Availability

The data used to support the findings of this study are made available upon reasonable request.
